# Hospital admissions from the emergency department of adult patients affected by myopathies

**DOI:** 10.1111/ene.16214

**Published:** 2024-01-16

**Authors:** Mauro Monforte, Eleonora Torchia, Sara Bortolani, Beatrice Ravera, Enzo Ricci, Gabriella Silvestri, Serenella Servidei, Guido Primiano, Massimiliano Mirabella, Mario Sabatelli, Eugenio Mercuri, Francesco Franceschi, Paolo Calabresi, Marcello Covino, Giorgio Tasca

**Affiliations:** ^1^ Dipartimento di Neuroscienze, Organi di Senso e Torace Fondazione Policlinico Universitario Agostino Gemelli IRCCS Rome Italy; ^2^ Università Cattolica del Sacro Cuore Rome Italy; ^3^ Centro Clinico NeMO Adulti Rome Italy; ^4^ Centro Clinico Nemo Pediatrico Fondazione Policlinico Universitario Agostino Gemelli IRCCS Roma Italy; ^5^ Pediatric Neurology Università Cattolica del Sacro Cuore Roma Italy; ^6^ Emergency Department Fondazione Policlinico Universitario A. Gemelli, IRCCS Rome Italy; ^7^ John Walton Muscular Dystrophy Research Centre Newcastle University Translational and Clinical Research Institute and Newcastle Hospitals NHS Foundation Trust Newcastle upon Tyne UK

**Keywords:** complications, emergency department, muscular dystrophies, myopathies

## Abstract

**Background and purpose:**

Myopathies are associated with classic signs and symptoms, but also with possible life‐threatening complications that may require assistance in an emergency setting. This phenomenon is understudied in the literature. We aimed to assess the presentation, management, and outcomes of clinical manifestations potentially related to a muscle disorder requiring referral to the adult emergency department (ED) and hospitalization.

**Methods:**

Anonymized patient data retrieved using the International Classification of Diseases, Ninth Revision codes related to muscle disorders over 4 years were retrospectively analyzed. Medical reports were evaluated to extract demographic and clinical variables, along with outcomes. Two groups were defined based on the presence (known diagnosis [KD] group) or absence (unknown diagnosis [UD] group) of a diagnosed muscle disorder at arrival.

**Results:**

A total of 244 patients were included, 51% of whom were affected by a known myopathy, predominantly limb‐girdle muscular dystrophies and myotonic dystrophies. The main reasons for ED visits in the KD group were respiratory issues, worsening of muscle weakness, and gastrointestinal problems. Heart complications were less prevalent. In the UD group, 27 patients received a new diagnosis of a specific primary muscle disorder after the ED access, mostly an inflammatory myopathy. Death during hospitalization was recorded in 26 patients, with a higher rate in the KD group and in patients affected by mitochondrial and inflammatory myopathies. Sepsis and dyspnea were associated with increased death risk.

**Conclusions:**

Respiratory complications are the most common reason for myopathic patients accessing the ED, followed by gastrointestinal issues. Infections are severe threats and, once hospitalized, these patients have relatively high mortality.

## INTRODUCTION

Myopathies are disorders primarily affecting the skeletal muscle, characterized by different signs and symptoms such as muscle weakness, cramps, stiffness, and pain. They can be caused by genetic defects or manifest as acquired forms, either isolated or in association with systemic diseases [1, 2, 3]. Cardiac or respiratory involvement [4] is also frequent in several myopathies. Inherited myopathies are usually slowly progressive, following a “chronic” course, with some exceptions, such as metabolic myopathies [5], which may be associated with acute events. In contrast, in acquired (inflammatory or toxic) myopathies, onset is often abrupt with rapid worsening. Acute muscle injury may be also caused by trauma or external factors, for example, necrotizing fasciitis, muscle abscess, or muscle hematoma. As a result, patients with various myopathies may seek medical assistance in an emergency setting, either for the relevance and progression of muscle symptoms or for possible life‐threatening cardiac and respiratory complications. However, despite the unexceptional occurrence and the multiple reasons for emergency department (ED) referral of myopathic patients, there is a lack of studies systematically analyzing the prevalence, characteristics, and outcomes of this phenomenon.

We designed this retrospective study to analyze the presentation, management, in‐hospital flow, and outcomes of clinical manifestations potentially related to a muscle disorder requiring referral to the ED and hospitalization in a large tertiary center. In particular, we wanted to: (i) assess the most frequent causes of ED access among patients already diagnosed with a muscle disorder, reflecting possible sudden worsening or complications of the known disease; (ii) explore the main health issues of patients who did not yet have a specific diagnosis; (iii) understand the most common new diagnoses of myopathies for patients admitted from the ED; and (iv) evaluate the outcomes and their main determinants in the different patient populations.

Awareness of the clinical picture and main causes leading to ED access and hospitalization of patients affected by muscle disorders can help prompt recognition. This audit of the workflow and outcomes from a tertiary referral center with longstanding experience is relevant to improving the medical care and in‐hospital management of this specific patient population.

## METHODS

Data were collected from adult (age >18 years) subjects hospitalized through the ED of the *Fondazione Policlinico Universitario Agostino Gemelli IRCCS* in Rome (Italy) between 2014 and 2018, to avoid any influence of the COVID‐19 pandemic. The study was conducted in accordance with the Declaration of Helsinki and its later amendments and was approved by the local institutional review board (IRB #0025817/22).

Consecutive cases were anonymously retrieved from the computerized clinical record database using the International Classification of Diseases, Ninth Revision (ICD‐9). All the codes in the 359 ICD‐9 group (“Muscular dystrophies and other myopathies”) and other relevant codes (such as 710.3 Dermatomyositis, 710.4 Polymyositis, 728.88 Rhabdomyolysis) were used for the query (full list detailed in Table S1).

For each case, the complete electronic medical record related to the ED visit and subsequent hospitalization was analyzed, collecting the following information: demographics, triage classification, vital parameters at arrival, chief and other complaints, serum creatine kinase level, previous medical history (diabetes, heart failure, vascular diseases, cancer, chronic obstructive pulmonary disease, dementia), ward of hospitalization, relevant procedures performed and disease course, length of stay, final diagnosis, and outcome. Patients were split into two groups: known diagnosis (KD) at admission (i.e., patients with a previously established diagnosis of myopathy), and patients with unknown diagnosis (UD). In the UD group, we further identified patients who eventually received a specific diagnosis of a muscle disorder during hospitalization (newly diagnosed [ND] subgroup).

Descriptive statistics are primarily used, and data are expressed as mean ± standard deviations, percentages, and medians with 95% confidence interval (CI). Differences in continuous variables were explored using the Mann–Whitney or Kruskal–Wallis test as appropriate. Differences in categorical variables were explored using Fisher's exact test or the chi‐squared test. Multivariable analysis of outcomes was performed modeling a multiple linear regression, with the length of hospitalization as the dependent variable and demographics, chief and other complaints, concomitant diseases, complications, and relevant procedures performed as predictors. A logistic regression, with survival as the dependent variable, was also modeled, using the same independent variables as previously reported. Statistical significance was set at *p* < 0.05, and all analyses were performed using IBM® SPSS Statistics v.28.

## RESULTS

**FIGURE 1:**
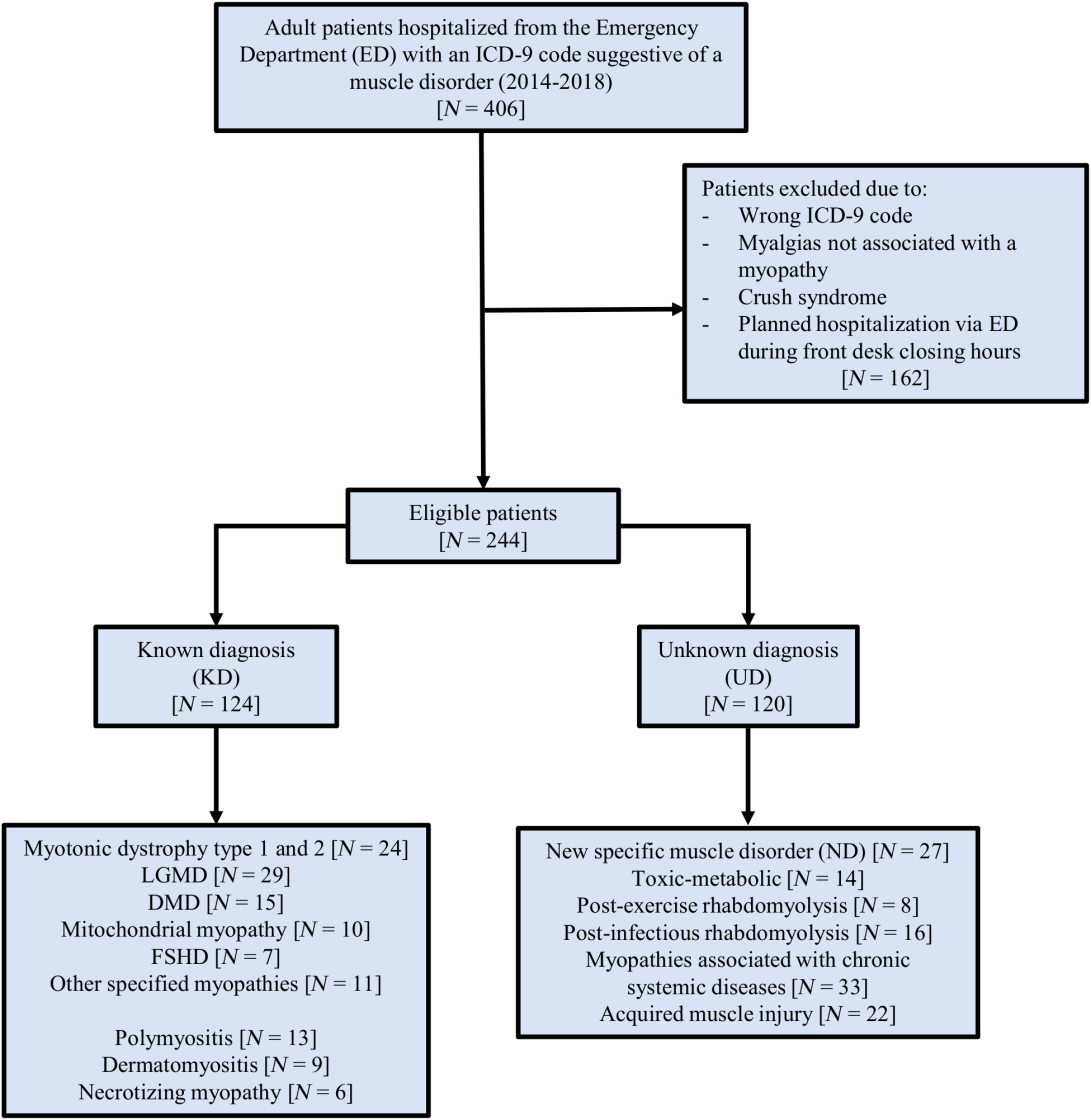
Flowchart of the patients included in the study. DMD, Duchenne muscular dystrophy; FSHD, facio‐scapulo‐humeral muscular dystrophy; ICD‐9, International Classification of Diseases, Ninth Revision; LGMD, limb‐girdle muscular dystrophy.

The total number of patients accessing the ED during the study period was 362,933, and 91,144 (25.1%) of these were hospitalized. Of these, 406 patients received an ICD‐9 code correlated with a muscle disorder, representing the initial query retrieval. After analysis of the medical records, 162 patients were removed for the following reasons: wrong ICD‐9 code attribution; myalgias not associated with myopathy (for example, chest pain in heart diseases); crush syndrome; and planned admission via ED during front desk closing hours. The final number of patients included in the study was 244 (137 male and 107 female; mean age 54.9 ± 19.2 years, range 18–89 years), representing 0.3% of the total hospital admissions from the ED (Figure 1). No seasonal trends or substantial yearly differences in the number of cases of ED access were identified, nor was any difference found between weekdays and weekends.

A total of 124 patients (51%) had an already established myopathy diagnosis (KD group), while 120 (49%) were undiagnosed at admission (UD group). Patients mainly reached the ED using their own means of transportation (*n* = 135, 55%), or by ambulance (*n* = 85, 35%). Two patients were transported using air ambulance. On a four‐point ED triage scale (White = non‐urgent, Green = deferrable, Yellow = urgent, Red = life‐threatening), 114 patients were coded Yellow (47%), 99 were coded Green (41%) and 21 were coded Red (9%). Patients were admitted to the following wards: 122 (50%) Internal Medicine and Cardiology, 97 (40%) Neurology, 14 (6%) Surgery and 11 (5%) Intensive Care Unit (Table 1).

**TABLE 1:** Comparison of the characteristics and outcomes of patients with and without a known diagnosis (KD) of a muscular disorder at emergency department arrival.

	KD group	UD group	*p* value
Sex, M:F	67:57	70:50	n.s.
Age, years	54 ± 20	56 ± 19	n.s.
Triage code, *n* (%)			
White	1 (0.8)	0 (0)	0.001
Green	35 (28)	64 (53)	0.001
Yellow	70 (56)	44 (37)	0.001
Red	12 (9.7)	9 (7.5)	0.001
CK at arrival, IU/L	1141 ± 1776	5629 ± 11,536	<0.001
CK difference at discharge	−1964 ± 2182	−4881 ± 10,395	n.s.
Diabetes, *n* (%)	11 (8.9)	13 (11)	n.s.
Vascular diseases, *n* (%)	13 (10)	17 (14)	n.s.
Cancer, *n* (%)	17 (14)	18 (15)	n.s.
Heart failure, *n* (%)	13 (10)	12 (10)	n.s.
COPD, *n* (%)	14 (11)	10 (8.3)	n.s.
Dementia, *n* (%)	5 (4)	1 (0.8)	n.s.
Abdominal disturbances, *n* (%)	21 (17)	12 (10)	n.s.
Dyspnea, *n* (%)	43 (35)	14 (12)	<0.001
Pneumonia, *n* (%)	29 (23)	12 (10)	<0.01
Sepsis, *n* (%)	15 (13)	8 (6.7)	n.s.
Other infections, *n* (%)	7 (5.6)	9 (7.5)	n.s.
Major surgery, *n* (%)	12 (9.7)	9 (7.5)	n.s.
Mechanical ventilation, *n* (%)	20 (16)	2 (1.7)	<0.001
Ward of hospitalization, *n* (%)			
Neurology	55 (44)	42 (35)	n.s.
Internal Medicine and Cardiology	55 (44)	68 (57)	
Intensive Care Unit	7 (5.6)	3 (2.5)	
Surgery	7 (5.6)	7 (5.8)	
Length of hospitalization, days	20.9 ± 31.3	16.2 ± 17.5	n.s.
Death	19 (15)	7 (5.8)	0.02

*Note*: Values expressed as mean ± standard deviation or number and %.

Abbreviations: CK, creatine kinase; COPD, chronic obstructive pulmonary disease; F, female; IU/L, international units per liter; KD, known diagnosis; M, male; n.s., nonsignificant; UD, unknown diagnosis.

The major cause for ED access in the KD group was respiratory (47 patients, 38%), either caused by primary respiratory muscle weakness (*n* = 28, 23%) or by a concomitant infectious disease (Table 2). Pneumonia was diagnosed in 14 patients (11%) and, in two patients, the lung infection was *ab ingestis*. Worsening muscle weakness was the reason for ED access in 21 patients (17%), followed by gastrointestinal causes (*n* = 18, 14%) and cardiac issues (*n* = 15, 12%). Abdominal pain associated with nausea and vomiting was observed mostly in patients affected by mitochondrial myopathies (*n* = 5, 45%) and dermatomyositis (*n* = 4, 44%). Regarding cardiac diseases, heart arrhythmia was the documented cause for ED access in seven patients of this group, mostly myotonic dystrophy type 1 or 2, and in four patients (three myotonic dystrophy type 1 and one facioscapulohumeral muscular dystrophy [FSHD]), the placement of an implantable pacemaker‐cardioverter defibrillator was necessary. In 15 patients (13%), mainly affected by inflammatory myopathies (*n* = 5) and myotonic dystrophy (*n* = 4), sepsis was documented and led to hospitalization after ED access, while four patients (3%) had a traumatic injury due to falls.

**TABLE 2:** Main cause for emergency department access in patients in the known diagnosis group.

	Patients, *N* (%)	Respiratory disease			Other specified causes, *n* (%)	Worsening of weakness *n* (%)	Gastrointestinal causes, *n* (%)	Heart disease		
		Respiratory failure, *n* (%)	Pneumonia, *n* (%)	Other, *n* (%)				Coronary heart disease, *n* (%)	Heart arrhythmia, *n* (%)	Heart failure, *n* (%)
Limb‐girdle muscular dystrophy	28 (23)	8 (27.6)	3 (10.3)	2 (6.9)	4 (13.8)	3 (10.3)	3 (10.3)	1 (3.4)	1 (3.4)	3 (10.3)
Myotonic dystrophy type 1 and 2	24 (19)	5 (20.8)	2 (8.3)	1 (4.2)	5 (20.8)	1 (4.2)	3 (12.5)	2 (8.3)	5 (20.8)	0 (0)
Duchenne muscular dystrophy	15 (12)	3 (20)	7 (46.7)	0 (0)	2 (13.3)	0 (0)	3 (20)	0 (0)	0 (0)	0 (0)
Polymyositis	13 (11)	5 (38.5)	0 (0)	1 (7.7)	3 (23.1)	4 (30.8)	0 (0)	0 (0)	0 (0)	0 (0)
Mitochondrial myopathy	11 (9)	2 (20)	1 (10)	1 (10)	1 (10)	0 (0)	5 (45.5)	0 (0)	0 (0)	1 (10)
Other specified myopathies	11 (9)	3 (27.3)	0 (0)	0 (0)	3 (27.3)	5 (45.5)	0 (0)	0 (0)	0 (0)	0 (0)
Dermatomyositis	9 (7)	1 (11.1)	0 (0)	0 (0)	3 (33.3)	0 (0)	4 (44.4)	0 (0)	0 (0)	1 (11.1)
Facioscapulohumeral muscular dystrophy	7 (6)	1 (14.3)	1 (14.3)	0 (0)	0 (0)	4 (57.1)	0 (0)	0 (0)	1 (14.3)	0 (0)
Immune‐mediated necrotizing myopathy	6 (4)	0 (0)	0 (0)	0 (0)	2 (33.3)	4 (66.7)	0 (0)	0 (0)	0 (0)	0 (0)
Total	124	47 (38)			23 (19)	21 (17)	18 (14)	15 (12)		

*Note*: Other specified causes: infectious diseases, sepsis, trauma, cancer. Other respiratory disease: chronic obstructive pulmonary disease, interstitial pulmonary fibrosis, pneumothorax.

The majority of patients in the KD group who accessed the ED were affected by limb‐girdle muscular dystrophy (LGMD; 29 patients, 23%), followed by myotonic dystrophy (*n* = 24, 19%) and Duchenne muscular dystrophy (DMD; *n* = 15, 12%). Among LGMD patients, by far the most prevalent reason for seeking urgent assessment and care was respiratory (*n* = 13, 45%), followed by cardiac issues (*n* = 5, 17%, mostly heart failure). Respiratory failure (*n* = 5, 21%) and heart arrhythmias (*n* = 5, 21%) were the main health issues for myotonic dystrophy patients, together with sepsis (*n* = 4, 17%). Three patients (13%) had major gastrointestinal complaints, in two cases this was due to a pseudo‐obstruction and in one case it was caused by a volvulus. Adult DMD patients instead had pulmonary infection in 47% of cases (*n* = 7), and less frequently had non‐infectious respiratory failure (*n* = 3, 20%) or gastrointestinal issues (*n* = 3, 20%).

In the group of immune‐mediated myopathies (dermatomyositis, necrotizing myopathy, or non‐otherwise specified inflammatory myopathy, i.e., polymyositis), worsening of muscle weakness and respiratory failure were the most prevalent causes, except for dermatomyositis patients, who mostly complained of serious gastrointestinal events. More specifically, two patients had nausea and vomiting due to drug‐induced hepatitis, one an acute diverticulitis and another a malignant bowel occlusion.

The majority of patients in the UD group at admission were later discovered to have an associated chronic systemic disease responsible for the muscle symptoms (*n* = 33, 28%), mainly kidney disease (eight patients), liver disease (eight patients), and heart failure (five patients). Twenty‐two patients (18%) were identified to have an acquired muscle injury (necrotizing fasciitis, muscle abscess, or hematoma). Sixteen patients (13%) had post‐infectious rhabdomyolysis. Fourteen patients (12%) had muscle signs and symptoms, either drug‐induced or associated with endocrine disorders (five cases of statin‐associated hyperCKemia, four cases of hyperCKemia from neuroleptic treatment, two cases of hypothyroid myopathy, two of colchicine‐induced myopathy and one of primary hyperaldosteronism). Eight patients (7%) had rhabdomyolysis after strenuous physical exercise. Finally, 27 (23%) were eventually diagnosed with a specific primary muscle disorder, either inflammatory or genetic (ND group). In this subgroup, an inflammatory myopathy was the most frequent diagnosis (23 patients, 85% of this group). Notably, one case of sporadic late‐onset nemaline myopathy [6] and one of myotonic dystrophy type 2 were also present, highlighting the possibility of a relatively acute onset or worsening of these rare conditions. The majority of patients in this group (*n* = 17, 63%) were triaged for muscle‐related symptoms (weakness, cramps, myalgias, rhabdomyolysis). Dysphagia (in four patients, two with inflammatory myopathies, one with sporadic late‐onset nemaline myopathy, and one with a mitochondrial myopathy) and respiratory failure (four patients, three of whom had inflammatory myopathies and one a congenital myopathy) were the other more frequent causes for ED access.

Creatine kinase dosage was available for 128 patients (52%). The average value at arrival was 4857 ± 10,651 IU/L (range 9–94,037 IU/L), with higher values for the UD group.

The mean (range) length of hospitalization (LOH) was 18.6 ± 25.5 (0.5–262) days, with no significant difference between the KD versus the UD group or among the distinct triage groups. The multiple linear regression model run to predict LOH was statistically significant (adjusted *R* ^2^: 0.355, *F*‐statistic: 26.221; *p* < 0.001). Major surgery (*β* = 16.9, 95% CI 12.3–21.1; *p* < 0.001), mechanical ventilation (*β* = 14.4, 95% CI 4.5–24.2; *p* = 0.04), sepsis (*β* = 21.0, 95% CI 11.3–30.7; *p* < 0.001) and pneumonia (*β* = 12.7, 95% CI 5.4–20.0; *p* < 0.001) significantly predicted longer LOH.

A total of 26 patients (11%) died during hospitalization (13 male and 13 female, mean [range] age 61.7 ± 17.3 [23–89] years), mainly from respiratory failure (*n* = 12, 50%) and sepsis (*n* = 7, 27%). The proportion of deceased subjects was significantly higher in the KD group versus the UD group (*p* < 0.02). The most frequent known diagnoses were mitochondrial myopathy (*n* = 5, 26%), polymyositis (*n* = 5, 26%), and LGMD (*n* = 4, 21%). The logistic regression model (death as dependent variable) was statistically significant (adjusted *R* ^2^: 0.129, chi‐squared = 16.0, *p* < 0.001, 88.5% of cases correctly classified) and showed that sepsis (odds ratio [OR] 5.4, 95% CI 1.9–15.6; *p* = 0.002) and dyspnea (OR 3.3, 95% CI 1.4–7.9; *p* = 0.007) increased the risk of death.

Although the percentage of patients who died was greater in the triage groups with higher priority codes (6% for Green, 11% for Yellow, and 29% for Red), this correlation was not confirmed in the multivariable analysis.

## DISCUSSION

In the present study, we provide an overview of the clinical presentation and outcomes of adult patients affected by muscle disorders who were hospitalized following an ED visit at our institution during a 4‐year period, independent from the bias of the COVID‐19 pandemic and therefore likely to represent a recent realistic scenario.

Our center serves as a reference for advanced healthcare and emergency treatment for a population of approximately 1.8 million inhabitants over a 4000‐km^2^ area to the north of the city of Rome and the Latium region (central Italy). Half of our study population was already diagnosed before ED admission and was mainly composed of LGMD, myotonic dystrophy, and DMD patients. The prevalence of the different forms of muscle disorders in the catchment area is assumed to have had an impact on these findings. Prevalence estimates in Italy vary widely: 1.7–3.4:100,000 inhabitants for DMD [7, 8], 9:100,000 for myotonic dystrophy type 1 [9], 1:100,000 for myotonic dystrophy type 2 [10], 5:100,000 for FSHD [11], and 0.5:100,000 for congenital muscular dystrophies [12], while the cumulative prevalence of all LGMDs was assessed to be up to 6.9:100,000 [13]. No specific data are available regarding the prevalence of immune‐mediated myopathies in Italy: an estimate from a systematic review set the worldwide prevalence to 14:100,000 inhabitants, although with relevant geographical differences [14]. For mitochondrial myopathies, the point prevalence reported in England in adults is 12.5:100,000 [15]. Even if exact data are not available, we can speculate that the cumulative prevalence of all the diseases considered in the present study is approximately 1000–1400 patients in the geographical area of interest and the annualized percentage of patients hospitalized from ED visits could then be estimated in the range 4.3%–6.1% based on our findings. This figure can be used as a reference by healthcare service providers when planning the resources needed to guarantee adequate care for these rare conditions, as well as by specialized neuromuscular centers to forecast the number of possible cases that may need urgent assistance.

Patients with LGMD were the most frequent users of our ED, despite LGMD not being the most common disease category. Our center has a longstanding tradition of managing such patients, and their attraction from neighboring regions may have contributed to the enrichment of these patients in our cohort. The second most frequent group was that of patients with myotonic dystrophy, due to the higher prevalence of these conditions but also to the frequent multi‐organ involvement and potential life‐threatening complications [16]. The relatively low number of patients with DMD may be attributable to the fact that a number of young adults are still often followed as part of a transition process in collaboration with the pediatric groups and may have different systems of referrals for direct admissions into inpatient wards without access to ED.

Of note, the main cause for ED referral and hospitalization in the KD group was respiratory insufficiency, sometimes associated (in 11% of cases) with a superimposed lung infection. This latter occurrence was particularly frequent in adult DMD patients accessing the ED, reaching up to almost half of the cases. Pneumonia is a common and severe complication of respiratory involvement in DMD patients, and interventions aimed at reducing this risk such as assisted coughing are recommended by current guidelines [17]. An increased alert for clinical signs of pneumonia (fever, respiratory distress, sputum production, hypoxemia), together with additional tests such as white blood cell count, C‐reactive protein concentration, and chest X‐ray, together with prompt antibiotic prescriptions, are relevant practices to be endorsed in the ED, aiming to provide better outcomes [18]. The occurrence of cases of pneumonia due to *ab ingestis* in our cohort highlights the importance of the assessment of swallowing difficulties in myopathic patients [19].

Gastrointestinal causes were also relatively frequent in our cohort (14% in the KD group), especially in patients affected by mitochondrial myopathies. It is estimated that more than 50% of the mitochondrial myopathies present with some form of enteric dysfunction, possibly caused by either central nervous system involvement, (autonomic) neuropathy or smooth muscle involvement in these multisystemic conditions [20, 21]. Acute intestinal pseudo‐obstruction represents the most severe complication of this spectrum, requiring prompt treatment [22]. Maybe more surprisingly, patients with dermatomyositis also had a relatively high rate of gastrointestinal complications, mainly as a consequence of immunosuppressive treatment or, of particular relevance, caused by a concomitant bowel malignancy. In 15%–30% of cases, dermatomyositis represents a paraneoplastic syndrome and the risk of cancer should be considered and screened accordingly in all patients, especially in specific serologic subgroups such as those that tested positive for anti‐transcription intermediary factor (TIF) 1‐γ or anti‐nuclear matrix protein (NXP) 2 autoantibodies [23].

A significant number of patients (13% of the KD group), especially those affected by inflammatory myopathies, had sepsis, and a contributor to this occurrence could be found in the use of immunosuppressive treatments to control the underlying muscle disorders.

Conversely, cardiac issues were among the least frequent causes of ED access in our cohort. Despite the relevant and invariable heart involvement in DMD [24], cardiac events were not at all recorded as the reason for ED access in these patients, highlighting the efficacy of currently implemented standards of care for cardiac surveillance and treatment [25]. However, cardiac assessment in the ED is strictly recommended in DMD as well as in other muscular dystrophies where the heart is involved. In myotonic dystrophy, for instance, the risk of a cardiac conduction disorder is 60 times higher than the population risk [26] and such patients have a threefold higher risk of sudden death, mostly of cardiac etiology. Interestingly, one FSHD patient had a symptomatic arrhythmia and needed the implantation of a pacemaker‐cardioverter defibrillator. Although severe cardiac involvement is exceptional in FSHD [27, 28], subclinical signs of (mainly arrhythmic) heart disease are probably more common [29, 30], and we cannot completely rule out the association between the muscle disorder and this event in our patient.

Finally, most of the patients known to be affected by inflammatory myopathies accessed the ED for relapses with acute or subacute worsening of muscle weakness.

Among the UD patients evaluated in the ED and then hospitalized for muscle‐related symptoms (hyperCKemia, weakness, cramps, myalgia), approximately one quarter received a subsequent diagnosis of specific primary muscle disorder, mainly an inflammatory myopathy, and a similar proportion was later diagnosed with a systemic disorder with secondary muscle involvement, mainly kidney and liver diseases. Chronic kidney disease is known to be associated with muscle damage, and the term “uremic myopathy” has been used to define this condition, which is characterized by muscle weakness and fatigue [31, 32]. Patients affected by liver diseases can also experience cramps, weakness or even rhabdomyolysis [33]. Our data underline that in these conditions muscle symptoms could sometimes develop acutely, leading to the need to seek emergency care.

Notably, three endocrine myopathies were also identified among the patients who presented to the ED for muscle‐related symptoms. These conditions are rare nowadays but should always be considered in an ED setting [34, 35], where routine assessment of blood pressure, serum electrolyte level, and thyroid function tests should be performed in patients who complain of weakness or cramps and myalgias and have raised CK levels.

Despite the overall low number of patients affected by myopathies admitted to our center across several years, which could be explained by overall effective management of these patients in the outpatient setting or with programmed hospitalization, the priority code assigned at triage indicates that approximately half of our population had health problems judged as minor, which could possibly have been managed in an outpatient setting. However, once Green‐triaged patients were hospitalized, their LOH was not different from that observed in patients triaged as having major health problems in the ED. This higher‐than‐expected admission rate for these patients with a theoretically mild condition may derive from the concern of the ED operator regarding the global frailty and substantial disability of subjects affected by muscle disorders, which are complex diseases needing high levels of care. This may have led to the decision not to discharge these patients and entrust them to other settings of care. In the future, we suggest that the empowerment of outpatient clinics and the implementation of at‐home settings of care, such as the application of telemedicine resources, could reduce patient exposure to non‐necessary risks, such as long stays, especially in ED waiting areas, and hospital infections. This could be a safer and more cost‐effective solution for these specific categories of patient. On the other hand, we observed a high mortality rate, up to 15% for the KD group. Respiratory failure and sepsis were the most common causes of mortality in this group, which was enriched with patients affected by mitochondrial myopathies and polymyositis. Patients with more urgent priority triage codes tended to have higher mortality, further reflecting the severity of the complication for which they were seeking emergency care.

We acknowledge, as limitations of this study, that some diseases may have been under‐ or miscoded by non‐neuromuscular experts. We also acknowledge that, given the presence of dedicated wards in our hospital, some patients with chronic diseases in follow‐up, such as DMD or mitochondrial disorders, but showing rapidly progressing worsening, may also be admitted directly to the ward without ED access. The generalizability of our results may also be limited because hospitalization rates can differ among hospitals/countries due to heterogeneity in the level of assistance guaranteed in various settings.

In conclusion, respiratory complications represent the most common reasons for ED referral and hospitalization of adult patients affected by muscle disorders and are among the leading causes of prolonged LOH and mortality. The information provided could be useful to clinicians and healthcare providers in adopting measures for appropriate care of these patients in the ED. Avoidance of long waiting times in the ED, prompt identification and treatment of infectious episodes, and referral to outpatient clinical care settings when possible are all interventions with the potential to improve outcomes in this population.

## AUTHOR CONTRIBUTIONS

**Marcello Covino:** Conceptualization; formal analysis; writing – original draft; methodology; supervision. **Mauro Monforte:** Conceptualization; formal analysis; writing – original draft; project administration; methodology. **Eleonora Torchia:** Data curation; formal analysis; writing – original draft. **Sara Bortolani:** Data curation; writing – review and editing; investigation. **Beatrice Ravera:** Data curation; investigation; writing – review and editing. **Enzo Ricci:** Writing – review and editing; investigation. **Gabriella Silvestri:** Writing – review and editing; investigation. **Serenella Servidei:** Writing – review and editing; investigation. **Guido Primiano:** Data curation; writing – review and editing. **Massimiliano Mirabella:** Writing – review and editing; investigation. **Mario Sabatelli:** Writing – review and editing; investigation. **Eugenio Mercuri:** Writing – review and editing; investigation. **Francesco Franceschi:** Writing – review and editing; investigation. **Paolo Calabresi:** Writing – review and editing; investigation. **Giorgio Tasca:** Conceptualization; formal analysis; writing – original draft; methodology; supervision.

## FUNDING INFORMATION

No funding was received for conducting this study.

## CONFLICT OF INTEREST STATEMENT

The authors have no conflicts of interest to declare that are relevant to the content of this article.

## Supporting information


Table S1:

## Data Availability

The datasets generated during and/or analyzed during the present study are available in anonymized form from the corresponding author on reasonable request.
