# Supplementary material for: Hospital admissions from the emergency department of adult patients affected by myopathies
Source: Eur J Neurol. 2024 Jan 16;31(5):e16214. doi: 10.1111/ene.16214 (PMC11236034; doi:10.1111/ene.16214)
Supplement: Supplementary file 1 — Table S1: [file ENE-31-e16214-s001.docx]

**Supplementary Table 1: list of the ICD-9 codes related to a muscle disorder used for the initial query of the study.**

[359](http://www.icd9data.com/2012/Volume1/320-389/350-359/359/359.htm) Muscular dystrophies and other myopathies

[359.0](http://www.icd9data.com/2012/Volume1/320-389/350-359/359/359.0.htm) Congenital hereditary muscular dystrophy

[359.1](http://www.icd9data.com/2012/Volume1/320-389/350-359/359/359.1.htm) Hereditary progressive muscular dystrophy

[359.2](http://www.icd9data.com/2012/Volume1/320-389/350-359/359/359.2.htm) Myotonic disorders

[359.21](http://www.icd9data.com/2012/Volume1/320-389/350-359/359/359.21.htm) Myotonic muscular dystrophy

[359.22](http://www.icd9data.com/2012/Volume1/320-389/350-359/359/359.22.htm) Myotonia congenita

[359.23](http://www.icd9data.com/2012/Volume1/320-389/350-359/359/359.23.htm) Myotonic chondrodystrophy

[359.24](http://www.icd9data.com/2012/Volume1/320-389/350-359/359/359.24.htm) Drug- induced myotonia

[359.29](http://www.icd9data.com/2012/Volume1/320-389/350-359/359/359.29.htm) Other specified myotonic disorder

[359.3](http://www.icd9data.com/2012/Volume1/320-389/350-359/359/359.3.htm) Periodic paralysis

[359.4](http://www.icd9data.com/2012/Volume1/320-389/350-359/359/359.4.htm) Toxic myopathy

[359.5](http://www.icd9data.com/2012/Volume1/320-389/350-359/359/359.5.htm) Myopathy in endocrine diseases classified elsewhere

[359.6](http://www.icd9data.com/2012/Volume1/320-389/350-359/359/359.6.htm) Symptomatic inflammatory myopathy in diseases classified elsewhere

[359.7](http://www.icd9data.com/2012/Volume1/320-389/350-359/359/359.7.htm) Inflammatory and immune myopathies

[359.71](http://www.icd9data.com/2012/Volume1/320-389/350-359/359/359.71.htm) Inclusion body myositis

[359.79](http://www.icd9data.com/2012/Volume1/320-389/350-359/359/359.79.htm) Other inflammatory and immune myopathies

[359.8](http://www.icd9data.com/2012/Volume1/320-389/350-359/359/359.8.htm) Other myopathies

[359.81](http://www.icd9data.com/2012/Volume1/320-389/350-359/359/359.81.htm) Critical illness myopathy

[359.89](http://www.icd9data.com/2012/Volume1/320-389/350-359/359/359.89.htm) Other myopathies

[359.9](http://www.icd9data.com/2012/Volume1/320-389/350-359/359/359.9.htm) Myopathy, unspecified

710.3 Dermatomyositis

710.4 Polymyositis

710.5 Eosinophilia myalgia syndrome

728.0 Infective myositis

728.1 Muscular calcification and ossification

728.10 Muscular calcification and ossification, unspecified

728.11 Progressive myositis ossificans

728.2 Muscular wasting and disuse atrophy, not elsewhere classified

728.3 Other specific muscle disorders

728.8 Other disorders of muscle ligament and fascia

728.81 Interstitial myositis

728.82 Foreign body granuloma of muscle

728.83 Rupture of muscle, nontraumatic

728.84 Diastasis of muscle

728.85 Spasm of muscle

728.86 Necrotizing fasciitis

728.87 Muscle weakness (generalized)

728.88 Rhabdomyolysis

728.89 Other disorders of muscle, ligament, and fascia

728.9 Unspecified disorder of muscle, ligament, and fascia

729.1 Myalgia and myositis, unspecified

074.1 Epidemic pleurodynia

271.0 Glycogenosis

277.87 Disorders of mitochondrial metabolism

791.3 Myoglobinuria
